# Foreign Body-Related Liver Abscess: A Case Study on Fishbone

**DOI:** 10.7759/cureus.60358

**Published:** 2024-05-15

**Authors:** Tram N Pham, Qui H Nguyen, Huong T Lam, Thang D Nguyen, Thong D Vo

**Affiliations:** 1 Internal Medicine, University of Medicine and Pharmacy at Ho Chi Minh City, Ho Chi Minh City, VNM; 2 Gastroenterology, Cho Ray Hospital, Ho Chi Minh City, VNM; 3 Gastroenterology, University Medical Center of Ho Chi Minh City (HCMC), Ho Chi Minh City, VNM

**Keywords:** antibiotic therapy, fishbone, foreign bodies, gastrointestinal perforation, liver abscess

## Abstract

Foreign body-related complications are rare but possibly fatal events in clinical practice. Liver abscess as a result of gastrointestinal perforation caused by foreign bodies is even more rare. We report a case of a 63-year-old man who was admitted with fever and left epigastric pain. Further investigation revealed a liver abscess without resolution despite antibiotic therapy for several weeks. In the second admission, an enhanced computerized tomography scan revealed multiple abscesses in the left lobe of the liver, with a linear radio-dense foreign body within the collection. Open surgery was performed to extract the foreign body. The patient made a satisfactory postoperative recovery without complications and was discharged on the sixth postoperative day.

## Introduction

A liver abscess as a result of a gastrointestinal perforation caused by a foreign body is extremely rare [1]. Because of relatively nonspecific symptoms and signs, in some cases, the diagnosis is only established by autopsy, and the mortality rate of cases with delayed definite diagnosis may be up to 100% [2]. Here, we present the case of a patient who had a liver abscess due to a fishbone, and it demonstrates the challenges in detecting a foreign body that is producing a liver abscess due to the lack of specific symptoms, the patient's forgetfulness of ingestion, and the low clinical suspicion of this condition.

## Case presentation

A 63-year-old male patient with a known medical history of hypertension was hospitalized due to the manifestation of high-grade fever, chills, anorexia, and overall malaise that had been persisting for the previous week. He reported no gastrointestinal manifestations, such as diarrhea, nausea, and abdominal discomfort, nor any respiratory indications, like coughing or sputum production. He affirmed his use of no alcohol, illicit substances, or tobacco products. Before presenting at an urgent care facility four days prior, he sought medical attention at a different healthcare facility for these issues. At the time, he was under treatment with cefotaxime and metronidazole on suspicion of a liver abscess, although he reported no improvement in his febrile conditions or general health status.

**Figure 1:**
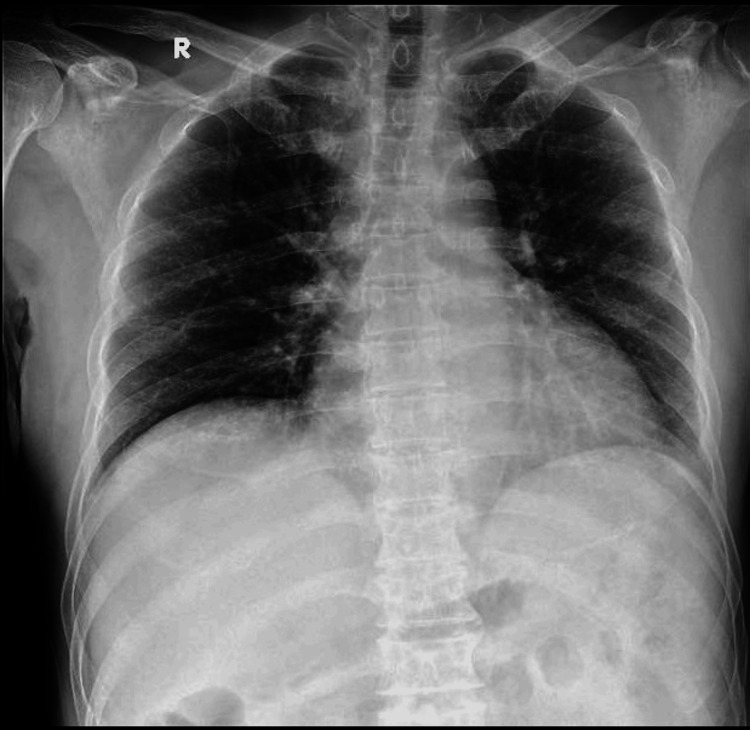
Chest radiograph showing an elevated right hemidiaphragm.

Upon clinical examination, the patient displayed alertness, and the recorded vital signs were a blood pressure of 130/60 mmHg, a heart rate of 76 beats per minute, and a respiratory rate of 20 respirations per minute. The patient's body temperature in the emergency department (ED) was recorded at 37°C. His oxygen saturation in room air was 98%. A cardiopulmonary auscultation revealed no abnormal findings. The abdominal examination revealed a soft, non-tender, non-distended abdomen with no discomfort on superficial or deep palpation and the absence of guarding or any indicators of peritoneal irritation. Laboratory findings are summarized in Table 1. The chest radiograph revealed an elevated right hemidiaphragm (Figure 1).

**Table 1:** Laboratory characteristics of the patient.

Laboratory tests	Result	Reference ranges
White blood cell (G/L)	10.66	4 to 11
Neutrophil (%)	78.5	45 to 75
Hemoglobin (g/L)	127	120 to 170
Platelets (g/L)	361	200 to 400
CRP (mg/L)	129.5	<6
Blood urea nitrogen (mg/dL)	16	7 to 20
Creatinine (mg/dL)	1.10	0.7 to 1.5
Aspartate aminotransferase (U/L)	40	9 to 48
Alanine aminotransferase (U/L)	38	5 to 49
Total bilirubin (mg/dL)	2.06	0.2 to 1
Direct bilirubin (mg/dL)	0.25	0 to 0.2
AFP (ng/ml)	1.0	<10
INR	1.1	1 to 1.2

**Figure 2:**
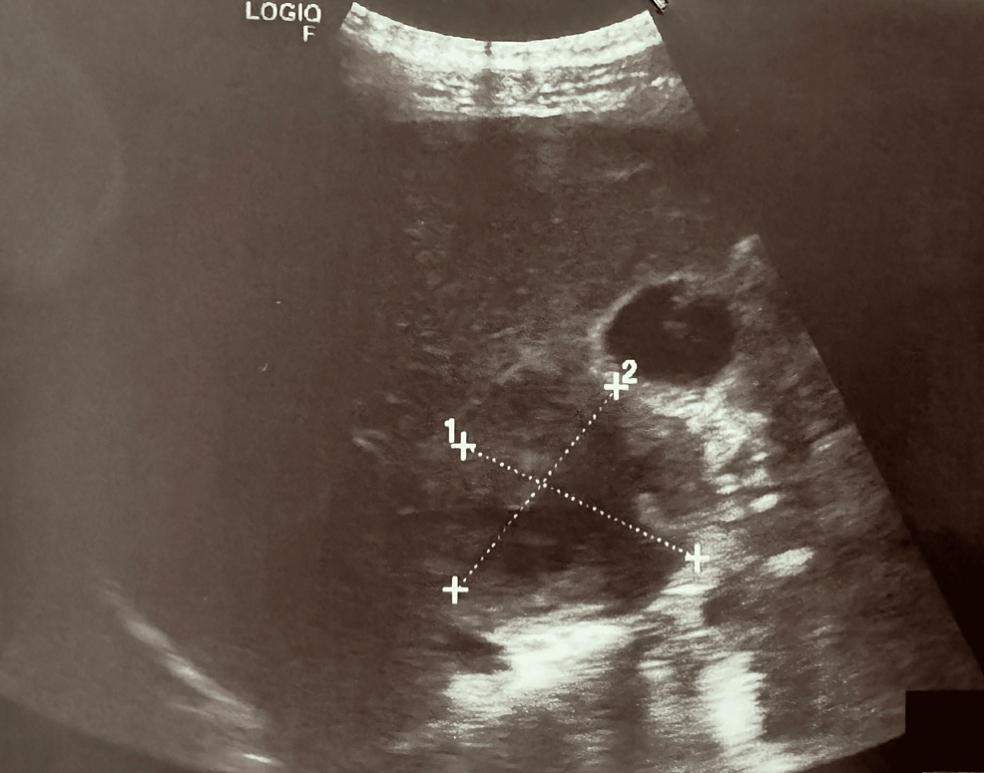
Liver abscess in the right lobe of the liver.

**Figure 3:**
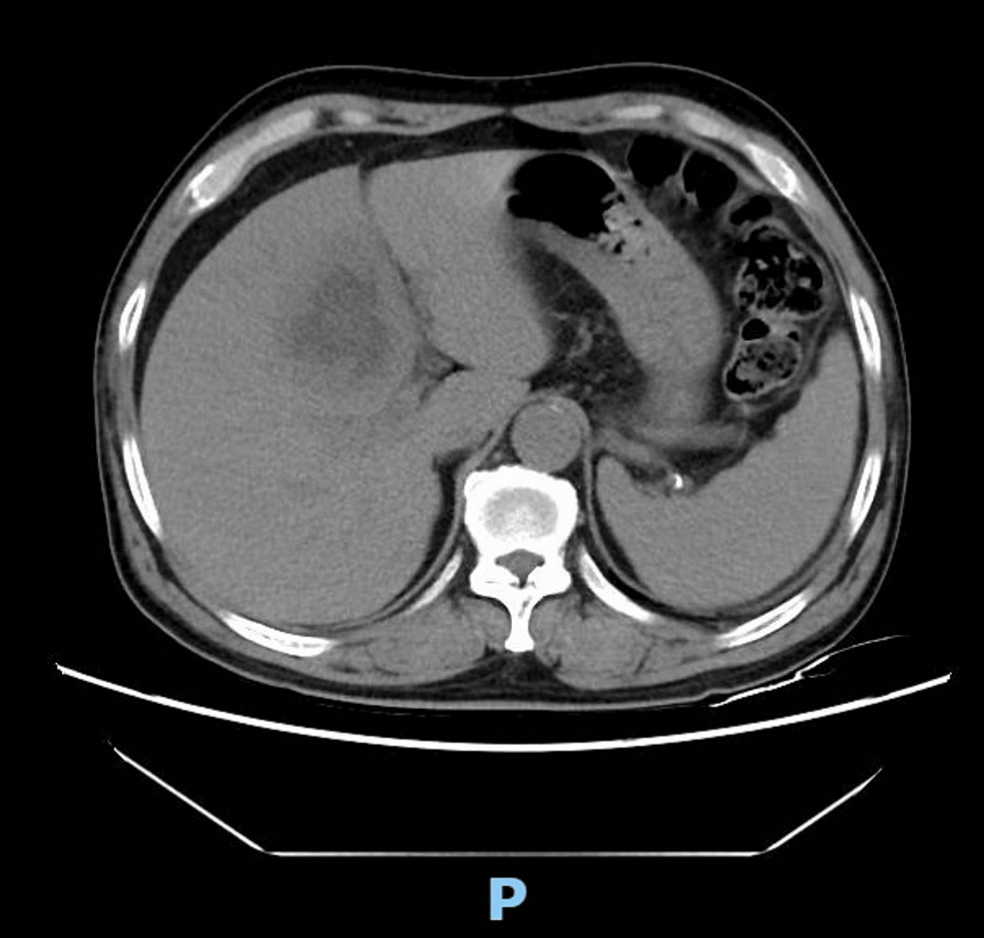
Abdominal computed tomography scan showing a hypodense lesion with irregular margins in segment IV (arrow).

Abdominal ultrasonography (US) disclosed a lesion with a 5cm diameter within the right lobe of the liver, as depicted in Figure 2. Subsequent diagnostic evaluation was conducted via a contrast-enhanced computed tomography (CT) scan of the abdomen and pelvis. This examination revealed a hypoattenuating lesion located in segment IV of the liver. The lesion, with a maximal diameter of 5 cm, exhibited peripheral enhancement, as indicated in Figure 3. Serological tests for Entamoeba histolytica and Fasciola proved to be negative.

Given the clinical presentation and imaging findings, the patient was diagnosed with a pyogenic liver abscess. An empiric intravenous regimen of metronidazole and ceftriaxone was initiated and continued for a 10-day duration. The patient exhibited a favorable response to the antibiotic therapy, with a resolution of fever and a decline in acute-phase reactants. Due to the marked clinical and biochemical improvement, he was discharged with an extended course of oral cefixime.

The patient returned to the hospital 35 days post-discharge, complaining of left epigastric pain and fever that had been present for the past two days. Upon admission, he was tachycardic, normotensive, and pyrexial, with a body temperature of 38.6°C. Physical examination revealed minimal epigastric tenderness, while the rest of the examination was within normal limits. Laboratory investigations revealed leukocytosis with a white blood cell count of 13.7 G/L, an International Normalized Ratio of 1.46, aspartate transaminase of 48 U/L, alanine transaminase of 43 U/L, and direct bilirubin of 0.85 mg/dl.

**Figure 4:**
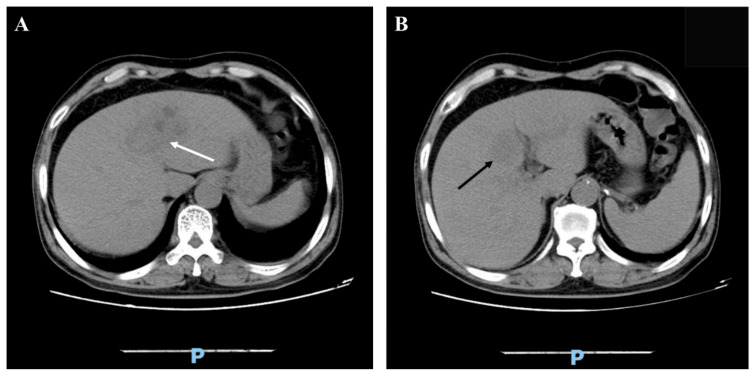
Abdominal computed tomography scan. A: A new abscess had developed in segment II (white arrow). B: The existing abscess in segment IV had decreased in size (black arrow).

**Figure 5:**
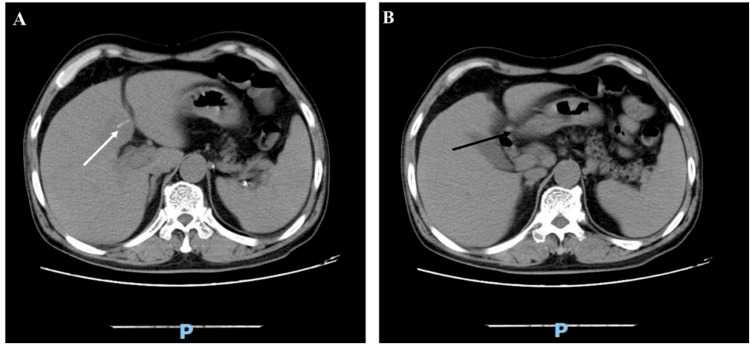
Abdominal computed tomography scan. A: A hyperdense linear body (white arrow) was found in the liver abscess. B: This linear body was attached to the stomach antrum (black arrow).

A computed tomography scan of the abdomen disclosed that the pre-existing abscess in liver segment IV had reduced in size. However, a new abscess had formed in liver segment II, as indicated in Figure 4. Within this new lesion, a high-density, 20 mm-long linear object was identified, adhering to the antrum of the stomach, as depicted in Figure 5. The medical team hypothesized that the patient might be dealing with a recurrent liver abscess due to a foreign body-induced gastric perforation. Consequently, a gastrointestinal endoscopy was performed, but the results were unremarkable.

A four-port laparoscopic procedure was initiated to drain the abscess and extract the foreign object from the patient. Inflammatory processes resulted in adhesions between the liver and the lesser omentum. Despite confirming the presence of purulent drainage and performing adhesiolysis, the foreign object could not be located laparoscopically. As a result, the surgical approach was converted to an open procedure.

**Figure 6:**
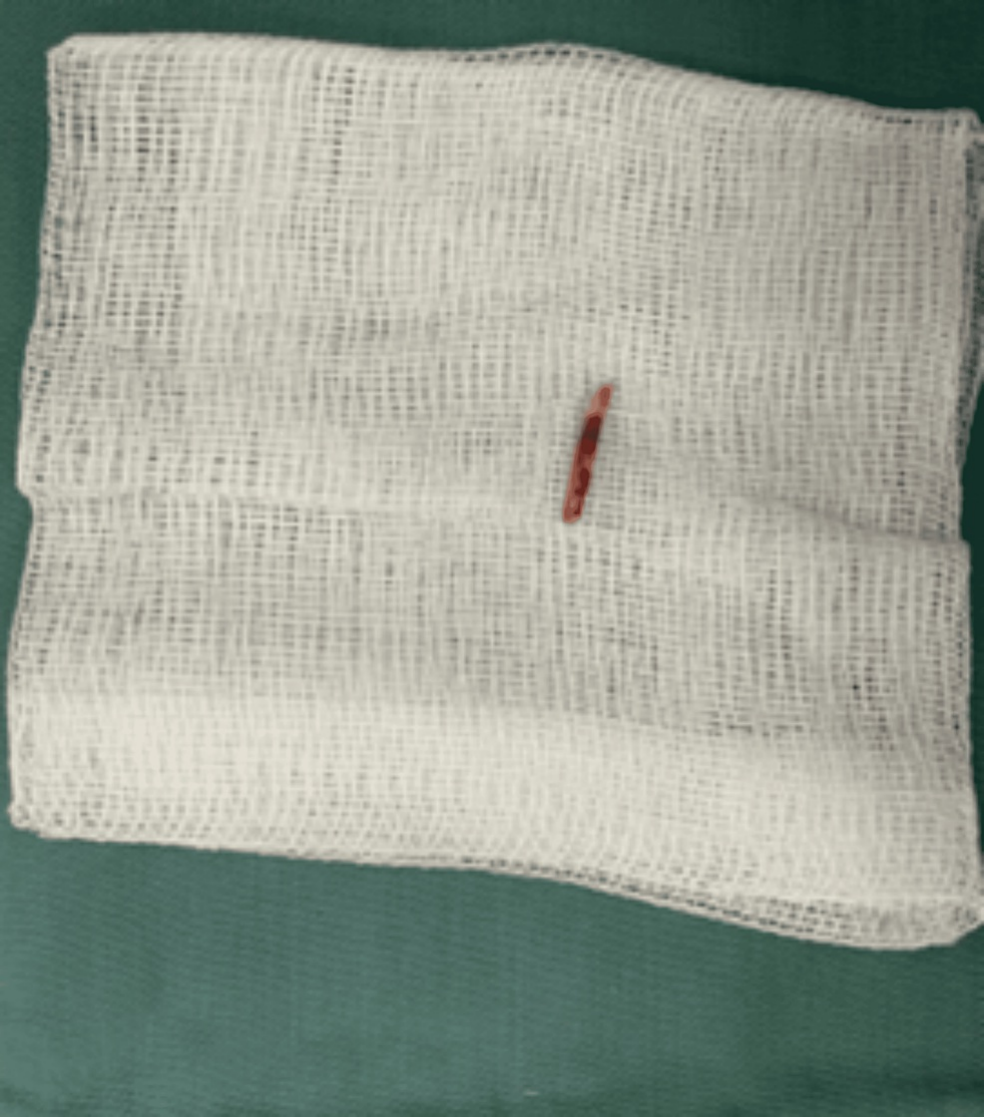
The image shows the fish bone discovered in the liver abscess.

Upon further exploration of the abscess, the foreign object was identified as a fishbone embedded in liver segment IV. The fishbone was successfully removed surgically, as illustrated in Figure 6. Subsequently, 300 ml of methylene blue was administered into the stomach via a Levin tube, and no leakage was observed. The abscess cavity was irrigated with saline, and closed-type drainage was implemented. A pus sample was collected for culture, which yielded no microbial growth. Concurrent blood cultures were also negative.

The patient was maintained on a regimen of metronidazole and piperacillin/tazobactam until the fifth postoperative day. The postoperative course was uneventful, leading to discharge on the sixth postoperative day. Upon follow-up one-month post-surgery, the patient was noted to have made a satisfactory recovery.

## Discussion

Most foreign bodies ingested pass through the gastrointestinal tract within one week [1]. Once swallowed, foreign bodies are often lodged in the oropharynx, such as in the tonsils, the base of the tongue, the vallecula, or the pyriform fossa [2]. Gastrointestinal perforation has been reported in less than 1% of cases, and the most common sites of perforation of the gut are the ileocecal and rectosigmoid regions [2]. Liver abscess, as a result of a gastrointestinal perforation caused by a foreign body, is even rare; the most common sites of perforation are the stomach and duodenum, which can be induced by sharp foreign bodies [3]. The first case was published by Lambert in 1898 [4]. Fishbone accounts for 33% of cases of liver abscess caused by a foreign body ingestion, followed by toothpick (27.3%) and chicken bone (12.5%) [5]. In Asian countries, since fish is commonly consumed, it is the most common foreign body that is ingested, as well as the one that poses the most significant risk for gastrointestinal perforation [6].

There are several case reports with the diagnosis of liver abscess due to foreign body only established by autopsy [4,7,8]. In a review of 88 liver abscesses due to foreign bodies, the mortality rate of those without definite diagnosis until autopsy was 100% (7/7) [5]. In this case, no cause for liver abscess was found at first admission, nor did the first abdominal contrast-enhanced CT scan reveal biliary tract abnormalities or foreign bodies. The patients did not remember swallowing the foreign body. No established imaging modality is the gold standard for evaluating the location and complications of the ingested foreign bodies [5,9]. Among the case reports of liver abscesses induced by foreign bodies, a CT scan remains the most commonly used imaging for the detection of foreign bodies [3,5]. Endoscopy may be helpful when performed early, before the foreign body migration and mucosal healing. In this case, when the liver abscess recurred after 35 days, the second CT scan detected the foreign body, but the gastrointestinal endoscopy was completely normal, showing that the mucosa had healed.

In previous case reports, the left lobe of the liver was the most common site of liver abscess due to foreign body, possibly because the most common sites of perforation are the stomach and duodenum [3,5,9]. This patient had an abscess at segment IV at the first admission. After 35 days, the CT scan of the abdomen revealed that the existing abscess in segment IV had shrunk in diameter, while a new abscess had developed in segment II of the liver.

In cases of liver abscesses induced by foreign body migration, the overall rate of cure without foreign body removal is low (9.5%) [5,10]. The overall mortality rate is 7.95%, and the major cause of death was septic shock [5]. The removal of the fishbone is the best way to treat and prevent the recurrence of the pyogenic liver abscess. It is possible to remove the foreign body through endoscopic or percutaneous removal in some cases [3]. When gastrointestinal endoscopy and laparoscopic surgery failed in this case, open surgery was the last option. In a review of 40 reported cases of liver abscess due to foreign body, open surgery was the method of foreign body removal in most cases [3].

## Conclusions

This case provides the literature with a rare case of pyogenic liver abscess caused by the ingested foreign body, which was cured. The diagnosis of liver abscess due to a gastrointestinal foreign body is difficult because of its rarity, nonspecific signs, and symptoms, and because the patient is often unaware of having swallowed the foreign body. The literature review showed that the delay in diagnosis causes poor patient outcomes. There is no gold-standard imaging for the diagnosis of pyogenic liver abscess induced by a foreign body, and imaging should be repeated in cryptogenic liver abscess cases with early recurrence. Surgery still plays a major role in the treatment of liver abscesses due to foreign body, although endoscopic or percutaneous foreign body removal is successful in some cases.
